# Cyanotoxins in Epipelic and Epiphytic Cyanobacteria from a Hypersaline Coastal Lagoon, an Environmental Hazard in Climate Warming Times and a Potential Source of New Compounds

**DOI:** 10.3390/md22080334

**Published:** 2024-07-24

**Authors:** Yerai Gómez-Leyva, Alejandro Torrecillas, Marina Aboal

**Affiliations:** 1 Laboratory of Algology, Faculty of Biology, Espinardo Campus, University of Murcia, E-30100 Murcia, Spain; yerai.gomez@um.es; 2 Service of Proteomics, CAID Building, Espinardo Campus, University of Murcia, E-30100 Murcia, Spain; alexts@um.es

**Keywords:** microcystin, saxitoxin, epipelon, epiphyton, Mediterranean coastal lagoons

## Abstract

Cyanobacterial biodiversity and potential toxicity in coastal lagoons have barely been studied despite these transitional water systems being very important in conservation and for the preservation of economic resources. Most of these transitional systems have been affected by eutrophication, and climate change will severely affect them by promoting cyanobacteria growth, especially in Mediterranean areas. This study aims to characterize the diversity of epipelic and epiphytic cyanobacteria species in a Mediterranean coastal lagoon and their potential for toxins production (microcystins and saxitoxins). Strains were isolated and genetically identified. Toxins were extracted and quantified by LC/MS-MS. All the taxa belong to the former Oscillatoriales. The presence of *Nodosilinea* and *Toxifilum* is reported for the first time for Spanish waters, but *Pseudanabaena, Phormidium*, *Geitlerinema* and *Synechococcus* also formed part of benthic mats. All the strains contained Microcystin-YR (MC-YR), but saxitoxin (STX) was present only in the extracts of *Nodosilinea* and *Pseudanabena*. MC-LY, MC-LW and [D-Asp^3^] MC-LR were detected in the extracts of *Synechococcus* and MC-LF in *Toxifilum*, but at concentrations that did not permit quantification. Toxins production by epipelic and epiphytic strains in coastal lagoons may represent a hazard, but also an opportunity to obtain potentially interesting compounds that should be further studied.

## 1. Introduction

Coastal lagoons are included in transitional waters and are characterized by their proximity to the sea and the influence of freshwater flows [1]. These water bodies are high-value ecosystems worldwide. They have been affected by several anthropogenic impacts [2], which must be added to the potential effects of climate warming. They are highly productive systems and support rich biodiversity [3].

Most coastal lagoons are shallow and are especially sensitive to external changes [4], but have been exploited by different human activities that have contributed to their degradation [5]. However, there is evidence that their communities are resilient to environmental changes and can buffer against external stresses under certain conditions [3,6].

Eutrophication has become evident in many of the world’s estuaries since the 1970s and is usually associated with diversity loss [7]. Nitrogen, rather than phosphorus, loadings are responsible for the perturbations produced by increased nutrients [8].

Brackish waters from the Mediterranean region remain relatively underexplored from the cyanobacterial point of view, but some authors have reported high abundances of pycocyanobacteria as *Synechococcus*-like in other geographic areas [9]. Even though the importance of this phytoplankton fraction in these systems has been normally neglected, its importance has been highlighted by metagenomic methods also in Mediterranean lagoons [10]. Freshwater cyanobacteria have been observed in brackish water worldwide, and *Microcystis aeruginosa* (Kütz) Kütz. is the most widely reported freshwater species in estuaries and demonstrates certain salt tolerance [11,12]. The transfer of microcystins from freshwater discharge to coastal environments has also been reported [13,14,15]. In most saline systems, however, photosynthetic benthic mats can be observed from sheltered coasts, estuarine habitats, salterns or hypersaline lagoons, and are usually composed of cyanobacteria, among other organisms [16,17]. Climate change will increase lagoon shallowness, which will favor not only the development of benthic communities, but also eutrophication levels to promote cyanobacterial growth [18].

Cyanobacteria’s ability to synthesize a plethora of secondary metabolites has increased the interest shown in their biotechnological applications and drug discovery [19,20,21]. Oscillatoriaceae may produce debromoaplysiatoxin, which is considered responsible for dermal toxicity and antileukemia activity [22,23]. Microcystin analogs are assumed to be selective anticancer drugs for those cancer types that express organic anion transporting polypeptides (OATPs), without any significant toxicity to normal cells because of differences in the redox status between normal and cancer cells [24]. Anatoxin-a (ATX-a) uses have been explored as mosquito larvicides [25,26] and saxitoxin (STX) reduces pain [27].

Hypersaline lagoons may contain mats with high taxa diversity regardless of the applied taxonomic approach [28]. Their biodiversity generally remains very poorly known from taxonomical and biochemical points of view. Recent floristic and taxonomic studies have shown that much work has to be done because many traditional genera are polyphyletic and are being split into several taxonomic entities [17,29,30,31,32,33].

This paper aims to characterize the cyanobacteria assemblages that live in the Mar Menor coastal lagoon and their potential to produce several cyanotoxins with different applied uses.

## 2. Results and Discussion

### 2.1. Morphological and Molecular Identification

**Figure 1:**
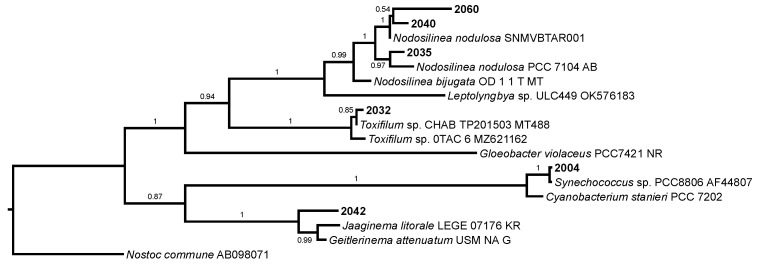
Bayesian topology showing the phylogenetic relations of the 16S rRNA gene sequence of the studied strains and 12 cyanobacterial strains using *Nostoc commune* AB098071 as an outgroup. Posterior probabilities are shown at consensus nodes.

**Figure 2:**
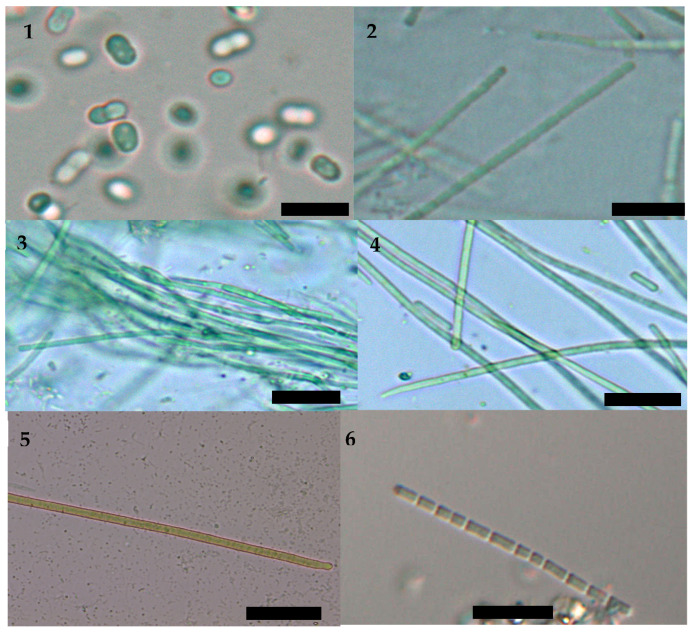
Cyanobacteria isolated from a Mediterranean coastal lagoon. **1***. Synechococcus* 2004, **2**. *Nodosilinea* 2035, **3**. *Toxifilum* 2032, **4**. *Geitlerinema* 2042, **5**. *Phormidium* 2058, **6**. *Pseudanabaena* 2122. The scale represents 20 μm.

The polyphasic taxonomy applied in this study revealed the presence in the lagoon of genera belonging to six different orders: *Synechococcus* (Synechococcales), *Nodosilinea* (Nodosilineales), *Toxifilum* (Occulatelales), *Pseudanabaena* (Pseudanabaenales), *Geitlerinema* (Geiterinematales) and *Phormidium* (Oscillatoriales). The identity of *Synechococcus*, *Nodosilinea* and *Toxifilum* was confirmed by sequencing, and the last two are reported for the first time in Spanish waters (Figure 1). As the sequencing of *Pseudanabaena, Geitlerinema* or *Phormidium* was not successful, we maintained their traditional taxonomic names based mainly on morphology until further molecular confirmations are available (Figure 2).

### 2.2. Cyanotoxins

**Figure 3:**
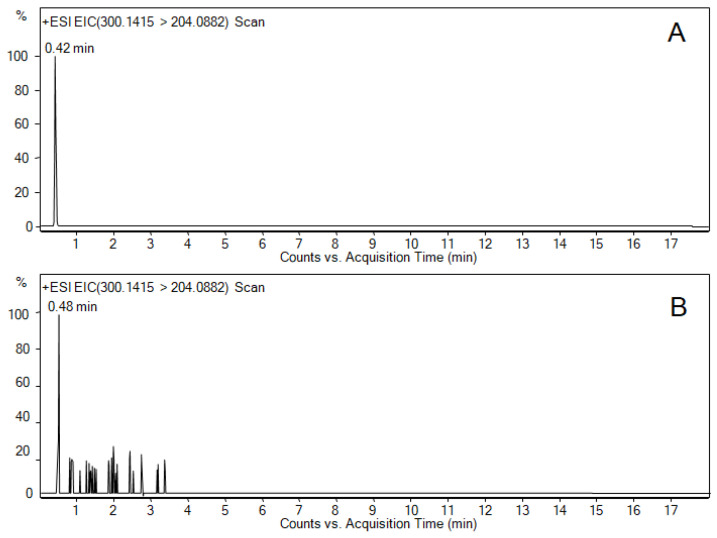

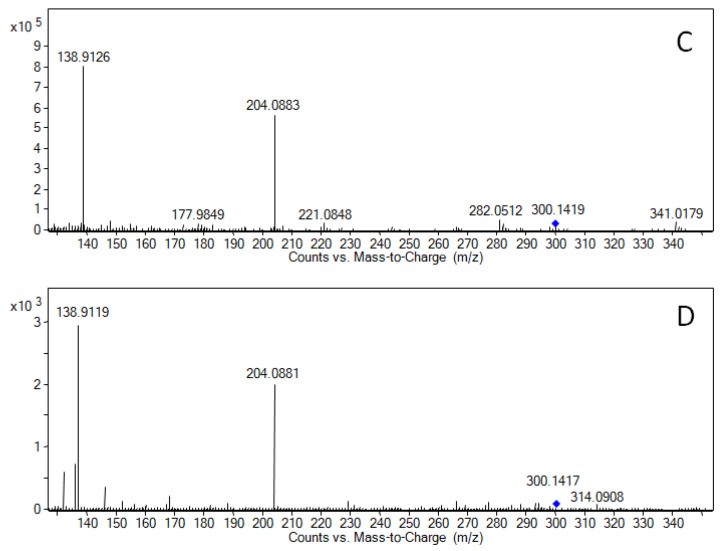
Extracted ion chromatograms of STX calibration standard (0.1 μg/mL) (**A**) and *Pseudanabaena* 21.22 sample (**B**). Mass spectra of the transition (300.1415 > 204.0882) corresponding to the STX in the calibration standard (**C**) and *Pseudanabaena* 21.22 sample (**D**). Collision energy of 60 eV was used for the fragment transition. Blue rhombus indicate the selected ion for transition analysis.

None of the isolated strains produced nodularin (NOD) or ATX-a at a detectable level. MC-YR was identified and quantified in the eight isolated strains (Table 1), and STX only in the *Toxifilum* and *Pseudanabaena* strains (Figure 3). MC-LF, MC-LY and [D-Asp^3^]MC-LR were identified in *Synechococcus*, but at such low concentrations that they remained below our method’s level of quantification (LoQ). The MC-LW concentration in the *Toxifilum* extract was also below the LoQ (Table 1).

The extracted ion chromatograms of STX and MC-YR calibration standards and *Pseudanabaena* 21.22 sample are shown in Figure 3 and Figure S1, as well as the mass spectra of the transition obtained with a collision energy of 60 eV.

Cyanotoxins producers were detected in epipelic and epiphytic mats in a coastal lagoon from Mediterranean SE Spain. It is very likely that the production of these compounds is much more frequent than presently thought in similar places. The environmental conditions of the Mar Menor coastal lagoon include lower salinity in the event of torrential rains close to the mouth of streams, high nitrogen compounds concentrations and a high temperature, which may promote cyanobacteria growth. This is the case of several other coastal lagoons worldwide. All these factors increase the potential of toxic events.

Cyanotoxins production by the inhabitants of these ecosystems has been very rarely reported. When the presence of toxins has been detected, they have been related mostly to the planktonic species that drift from close freshwater water bodies [11,14]. However, growing interest is being shown in marine benthic mats and their capacity to produce toxins and bioactive compounds in warm areas [32,34,35].

The cyanobacterial biodiversity of these Mediterranean transitional water bodies has been very poorly studied. Despite some attention having been recently paid to them in other areas [14,36,37,38], most data refer to the abundance of former Oscillatoriales and Nostocales [37]. The taxonomy of these two orders has vastly changed lately to include a good number of new genera, some of them by splitting old polyphyletic ones [39,40,41]. The importance of a polyphasic approach for the study and identification of cyanobacteria must be emphasized to obtain a more realistic biodiversity database. More often than not, data interpretations and comparisons are presently extremely difficult.

Subtropical and tropical regions have been intensely studied recently and a good number of new taxa have been described, which demonstrate that biodiversity of these areas is probably much higher than presently known [30].

*Microcystis* has been reported from transitional waters, where it can survive at a relatively high temperature and salinity, and toxicity is maintained, but reduced [42]. However, some authors reported relatively high concentrations of MC-LR in a coastal lagoon from Northern Africa [43] This genus was not collected in the studied lagoon, probably because there are no water bodies where it can grow in the basin.

In our samples, *Nodosilinea* was frequent, and its diversity is now under study. The genus has been described from freshwater habitats [29]. However, it has a wide ecological range and geographical distribution after being recently reported from terrestrial habitats in Antarctica [44], cold deserts [45], saline alkaline lakes [46] and mangroves, or after having been associated with marine sponges [47]. Under our experimental conditions, some, but not all, strains produced their characteristic nodules, as other authors have previously reported [48].

Our *Nodosilinea* strains produce MC-YR and STX. Gkelis et al. [48] did not find any toxins in *Nodosilinea* or *Synechococcus* in Greek freshwater. Leao et al. [49] reported lethality to *Artemia salina* in aqueous extracts in free-living forms from *Nodosilinea* but did not refer to its toxic compounds. The *Nodosilinea* and *Synechococcus* strains isolated from diverse habitats were tested against cancer cell lines. They have strong cytotoxicity effects [50], but bioactive compounds are not mentioned.

*Toxifilum* may also produce very conspicuous growth as in the lagoon from where it has been described [31]. Its toxicity is reported from its description, and it may produce Anabaenopeptin G. The possibility of its production in these localities should be investigated in the future.

The concentration values of microcystins in samples studied are similar in other European coastal areas [14]. The high frequency of MC-YR has already been detected in other localities from SE Spain [51]. A European continental study has shown that its increased frequency is related to a rise in temperature [52].

STX production by benthic cyanobacteria has been previously assessed in several Oscillatoriales [53,54,55,56,57]. Zupancic et al. [58] reported the potential of this toxin to synthesize with quantitative PCR in biofilms. Borges et al. [54] also confirmed that *Geitlerinema* and *Phormidium* from sediments may produce several different STXs in the reservoirs they sampled in a semiarid region of Brazil and our concentration values are among ranges reported by WHO [59].

*Phormidium*, *Leptolyngbya* and *Geitlerinema* also produce several toxins in Southern California [37], and the authors mentioned the frequency of multiple cyanotoxins producers in that area, where the three genera are very common. The co-occurrence of toxins in a water sample, which was once considered a rare phenomenon, seems much more frequent than originally assumed, and cyanobacterial assemblages contained multiple potential toxin producers at each studied site [37].

The fact that the concentration of some microcystin (MC) variants in our samples was below the LoQ implies that further in-depth studies are necessary with field and cultured materials, especially when cyanobacteria communities of similar species composition are common along Mediterranean coasts [60].

The potentiality of anticancer compounds production has been reported for all the collected taxa [50], but they may also generate anti-inflammatory, antibacterial or antiplasmodial compounds [61,62]. Saxitoxin is drawing much interest for its potential application in pain control, among other applications [27,63].

Climate change models usually show the future increase in temperature and nutrients in continental waters. Both factors promote cyanobacteria growth by increasing the probability of harmful events and the need for further studies. Nonetheless, cyanobacteria may also be beneficial by contributing to water bioremediation and producing chemical compounds that might provide pharmaceutical and medical uses.

## 3. Conclusions

Coastal lagoons may represent a source of compounds of biotechnological interest especially in a context of climatic changes leading to an increase of temperature and eutrophication that favor the growth of cyanobacteria.

Epipelic and epiphytic communities are very likely much more diverse than known and should be more intensely studied.

The possibility to identify and quantify several chemically different toxins at the same time will facilitate monitoring of the very frequent multitoxic samples in all aquatic systems, especially in a context of climatic change.

## 4. Materials and Methods

### 4.1. Study Area

The Mar Menor is one of the largest hypersaline coastal lagoons of the Mediterranean Sea. It covers 135 km^2^ and its mean depth is 4.5 m. Water temperature ranges between 24.5 °C and 30 °C. Salinity, which was formerly around 74 mS/cm, now lies at 63.6–68.9 mS/cm, with pH between 7.6 and 8.2 [64]. As the agriculture surrounding the lagoon has extended, several freshwater ephemeral watercourses drain in it and have become permanent streams, which increases the nitrate load and reduces the salinity in the vicinity of their mouths [61].

### 4.2. Sampling

Sampling was undertaken in spring 2018. Epipelic samples were collected with a spatula from seven sites in the shallowest lagoon areas, close to mouth of streams (Table 2) where mud accumulates. *Cymodocea* plants were collected by hand from shallow meadows, and leaves were squeezed to recover epiphytes. All the samples were placed inside plastic bags and transported to the laboratory in a portable cooler. The aliquots of each sample were fixed in acetic Lugol (5%). Localities are indicated in Table 2, along with their habitat, depth, conductivity ranges and main nutrient concentrations.

### 4.3. Isolation and Culture

The aliquots of the field samples were vortexed (1 min) for trichomes separation purposes and placed on a Petri dish (6 cm diameter) with several drops of Gillard f2 medium [65] prepared with filtered water from the lagoon. The isolated trichome was transferred with a Pasteur pipette (under an inverted microscope) until one trichome was left in a drop. The isolated trichome was first transferred to a 9 cm-diameter Petri dish with the same, but agarized, medium, and was placed inside culture flasks when growth was observed [54]. Flasks and dishes were incubated at 20 °C and 80 μmol photons m^−2^ s^−1^ with a 16 h:8 h L:D photoperiod.

The cultivation period lasted 1–2 months to obtain enough biomass for the toxin analyses and DNA extractions. The biomass was recovered by centrifugation and was frozen at −80 °C and/or lyophilized. The lyophilated biomass was stored at −20 °C.

### 4.4. Morphological Taxonomic Identification

Morphological taxonomic identification was done following Anagnostidis and Komarek’s [66,67] criteria (trichome and filament diameter, trichome constrictions, cell diameter and length, motility, apical cell morphology). The nomenclature was updated according to [39,40,41]. At least 20 trichome dimensions were measured for each strain under an Olympus BX50 microscope with a digital camera and the *Soft Imaging System ColorView* I software (CellSens V3.2).

### 4.5. DNA Extraction, Sequencing and Data Analysis

DNA was extracted from the cultured material by the CTAB method [68]. 16S PCR amplifications were carried using CYA106F as the forward primer and WAW1480 as the reverse primer [29,69]. Reactions were run in a total volume of 25 µL, consisting of 5.2 µL MyTaq™ reaction buffer, 0.7 µL 10 µM forward and reverse primers, 0.125 µL 1 U/µL My Taq™ DNA Polymerase (Bioline, Mumbai, India), 17.475 µL MilliQ^®^ water and 1 µL template DNA. PCR was carried out with initial denaturation at 94 °C for 5 min, followed by 25 denaturation cycles at 94 °C for 50 s, annealing at 60 °C for 50 s, and extension at 72 °C for 1 min and 30 s, with a final extension at 72 °C for 10 min. PCR products were purified employing ExoSAP-IT^®^ (Bell 2018) and sequenced by Macrogen (Seoul, Republic of Korea).

Sequences were edited using Bioedit 5.0.9 [69] and aligned with CLUSTALW [70] with minor manual adjustments. Data were analyzed by Bayesian inference, implemented with MrBayes 3.7 [71,72,73] using *Nostoc commune* (AB098071) as an outgroup and the reference sequences taken from CyanoSeq [74] and the NCBI database. Trees were sampled across the substitution model space in the Bayesian MCMC analysis [75] by applying the nst = mixed option. Therefore, a priori model testing was not necessary. Two runs with four chains were conducted with 2,000,000 generations. Trees were sampled every 1000th generation and the first 500,000 generations were discarded (burn-in) to exclude trees before the chain reached the stationary phase. The following checks were done: the stationarity of the log likelihood values; if the Potential Scale Reduction Factor (PSRF) came close to “1” (0.99 < PSRF < 1.01); if the estimated sample size was above 300 for all the parameters. The final trees were edited with TreeGraph2 [76].

### 4.6. Toxins Extractions

Extractions were done from cyanobacterial biomass according to [77,78]. Samples were transferred to 10 mL glass tubes and maintained in a freeze-drier for 4 h. MCs were extracted 3 times at 45 °C in 2.5 mL 75% methanol-25% Millipore water (*v*/*v*). Extracts were dried in a Speedvac (Savant SPD 121P, Waltham, MA, USA) and reconstituted in 900 μL methanol. The reconstituted samples were transferred to 2 mL Eppendorf vials with a cellulose-acetate filter (0.2 μm, Corning Costar Spin-X centrifuge tube filters) and centrifuged for 5 min at 16,000× *g* (Optima L-100 xp Beckman Coulter, Brea, CA, USA). Filtrates were transferred to amber glass vials for the LC-MS/MS analysis.

### 4.7. LC-MS/MS Analysis

Samples were analyzed for seven MC variants (MC-LR, MC-RR, MC-YR, MC-LF, MC-LW, MC-LY, D-Asp3-MC-LR), STX, ATX-a and NOD by LC-MS/MS as described in [76] with some modifications.

The HPLC-ESI-MS/MS analysis was performed in an Agilent 1290 Infinity Series II LC (consisting in a 1290 high-speed binary pump module G7120A, a 1290 Multisampler module G7167B and a 1290 Multicolumn thermostat module G7116B) and in an Agilent 6550B Q-TOF equipped with an Agilent JetStream Dual-Electrospray (ESI) and an i-Funnel. Compounds were separated in an Agilent Eclipse Plus C18 2.1 × 100 mm, 1.8 μm column, and were thermostatted at 40 °C using MilliQ water with 0.1% formic acid (*v*/*v* eluent A) and acetonitrile with 0.1% formic acid (*v*/*v*, eluent B). The elution program was 0–2 min 30% B, 2–6 min with a linear increase from 30 to 90% B, 5 min at 90% B, and a 5 min postrun at 30% B. The solvent flow rate was 0.5 mL/min and the injection volume was 20 μL.

LC-MS/MS operated in the positive mode, and nitrogen was used as the drying and collision gas. The drying gas temperature was set at 130 °C, with a flow of 16 l/min and 30 psi nebulizer pressure. The sheath gas temperature and flow were set at 300 °C and 11 l/min, respectively. The capillary spray, nozzle, fragmentor and octopole 1 RF Vpp voltages were 4000 V, 500 V, 360 V and 750 V, respectively. The centroid data within the 100–1700 *m*/*z* range were acquired for the MS scans in the 2 GHz Extended Dynamic Range High Resolution mode with four spectra, the 250 ms/spectrum and the 2026 transients/spectrum. Reference masses at 121.0509 and 922.0098 *m*/*z* were used for mass correction during the analysis. Three different collision energies (0, 10 and 40 V) were set for fragmentation confirmation. Data analyses were performed with the MassHunter Qualitative Analysis Navigator software (Agilent Technologies, Rev. B.08.00, Santa Clara, CA, USA). The extracted ion chromatograms of the compounds in Table 3 were analyzed. Calibration standards were obtained from Abraxis and prepared in methanol (Figure 3). Samples were quantified against a calibration curve and subsequently corrected for recovery. Each sample was injected once.

As the retention time for STX (0.42 min) was very close to the dead volume column time indicated in the certificate of analysis provided by the manufacturer (0.417 min), we confirmed the identification of this compound using the exact mass MRM transition of a standard (0.1 μg/mL) and positive samples using a collision energy of 60 eV (Figure 3). Both exact mass and fragment transition confirm the presence of STX [79].

## Figures and Tables

**Table 1:** Cyanotoxins detected and quantified in the Mar Menor coastal lagoon (LoQ = level of quantification).

Strain	MC-YR μg/g	MC-LF μg/g	MC-LW μg/g	MC-LY μg/g	[D-Asp3] MC-LR μg/g	STX μg/g
*Synechococcus* 2004	0.56	<LoQ	-	<LoQ	<LoQ	-
*Toxifilum* 2032	22.22	-	<LoQ	-	-	29.63
*Nodosilinea* 2035	6.63	-	-	-	-	-
*Nodosilinea* 2040	3.68	-	-	-	-	-
*Geitlerinema* 2042	6.06	-	-	-	-	-
*Nodosilinea* 2060	3.43	-	-	-	-	-
*Pseudanabaena* 2122	11.44	-	-	-	-	21.45
*Phormidium* 2058	1.20	-	-	-	-	-

**Table 2:** Sampling sites with indications of habitat, depth, conductivity ranges, and nitrate and orthophosphate concentrations [64].

Sampling Point	Localities	Habitat	Depth (cm)	Conductivity mS/cm	NO_3_ mg/L	PO_4_ mg/L
**1**	Breakwater Punta Brava, Los Urrutias	Epiphyte *Cymodocea*	20	30–48.2	65.8–68.3	1.2–2.2
**2**	Mouth Rambla Albujón, Los Narejos	Epipelic	5	35–62	87.1–103.2	<0.01–12.8
**3**	Mouth Rambla Miranda, El Carmolí	Epipelic	5	28–40	68.9–74.2	1.6–3.4
**4**	Mouth Rambla del Miedo, Los Urrutias	Epipelic	8	30–75	68.8–86.1	<0.01–1.1
**5**	Mouth Rambla Fangal, Cartagena Port	Epipelic	10	56–57	28.6–49.9	<0.01
**6**	Rambla de Benipila, Algameca Chica	Epipelic	10	56–57	21.4–64.2	<0.01
**7**	Molino Derribado, San Pedro del Pinatar	Epiphyte *Cymodocea*	25	25–49.5	48.9–76.5	0.8–1.9
**8**	Villananitos, San Pedro del Pinatar	Epiphyte *Cymodocea*	20	25–49.5	60.1–61.3	0.6–2.2

**Table 3:** Compounds analyzed during the LC-MS/MS analysis with their LoQ, LoD and linear range.

Compound	Formula	*m*/*z*	MS/MS	RT (min)	LoD (μg/mL)	LoQ (μg/mL)	Linear Range (μg/mL)
ATX-a	C_10_H_15_NO	166.1241	130.0498	0.66	0.001	0.004	0.001–1
STX	C_10_H_17_N_7_O_4_	300.1415	204.0882	0.42	0.0001	0.0005	0.0001–0.25
MC-RR	C_49_H_75_N_13_O_12_	519.7902 (+2)	213.0875	1.25	0.001	0.005	0.001–2.5
NOD	C_41_H_60_N_8_O_10_	825.451	135.0805	2.46	0.001	0.003	0.001–5
[D-Asp^3^] MC-LR	C_48_H_72_N_10_O_12_	981.5404	135.0805	3.31	0.0001	0.0005	0.0001–0.5
MC-LF	C_52_H_71_N_7_O_12_	986.5233	164.9845	4.89	0.001	0.004	0.001–0.75
MC-LR	C_49_H_74_N_10_O_12_	995.5567	599.3553	3,33	0.0005	0.002	0.0005–1
MC-LY	C_52_H_71_N_7_O_13_	1002.5183	213.0875	4.44	0.001	0.005	0.001–5
MC-LW	C_54_H_72_N_8_O_12_	1025.532	517.2761	4.78	0.001	0.005	0.001–5
MC-YR	C_52_H_72_N_10_O_13_	1045.5316	135.0804	3.18	0.001	0.004	0.001–5

## Data Availability

All the data will be sent by the authors upon request.

## Supplementary Materials

The following supporting information can be downloaded at: https://www.mdpi.com/article/10.3390/md22080334/s1. Figure S1. Extracted ion chromatograms of MC-YR calibration standard and Pseudanabaena 21.22 and mass spectra of the transition (1045.5316 > 135.0804) corresponding to the MC-YR in calibration standard and Pseudanabaena 21.22.
