# Supplementary material for: Cyanotoxins in Epipelic and Epiphytic Cyanobacteria from a Hypersaline Coastal Lagoon, an Environmental Hazard in Climate Warming Times and a Potential Source of New Compounds
Source: Mar Drugs. 2024 Jul 24;22(8):334. doi: 10.3390/md22080334 (PMC11355275; doi:10.3390/md22080334)
Supplement: Supplementary file 1 [file marinedrugs-22-00334-s001.zip › marinedrugs-3073261-supplementary.pdf]

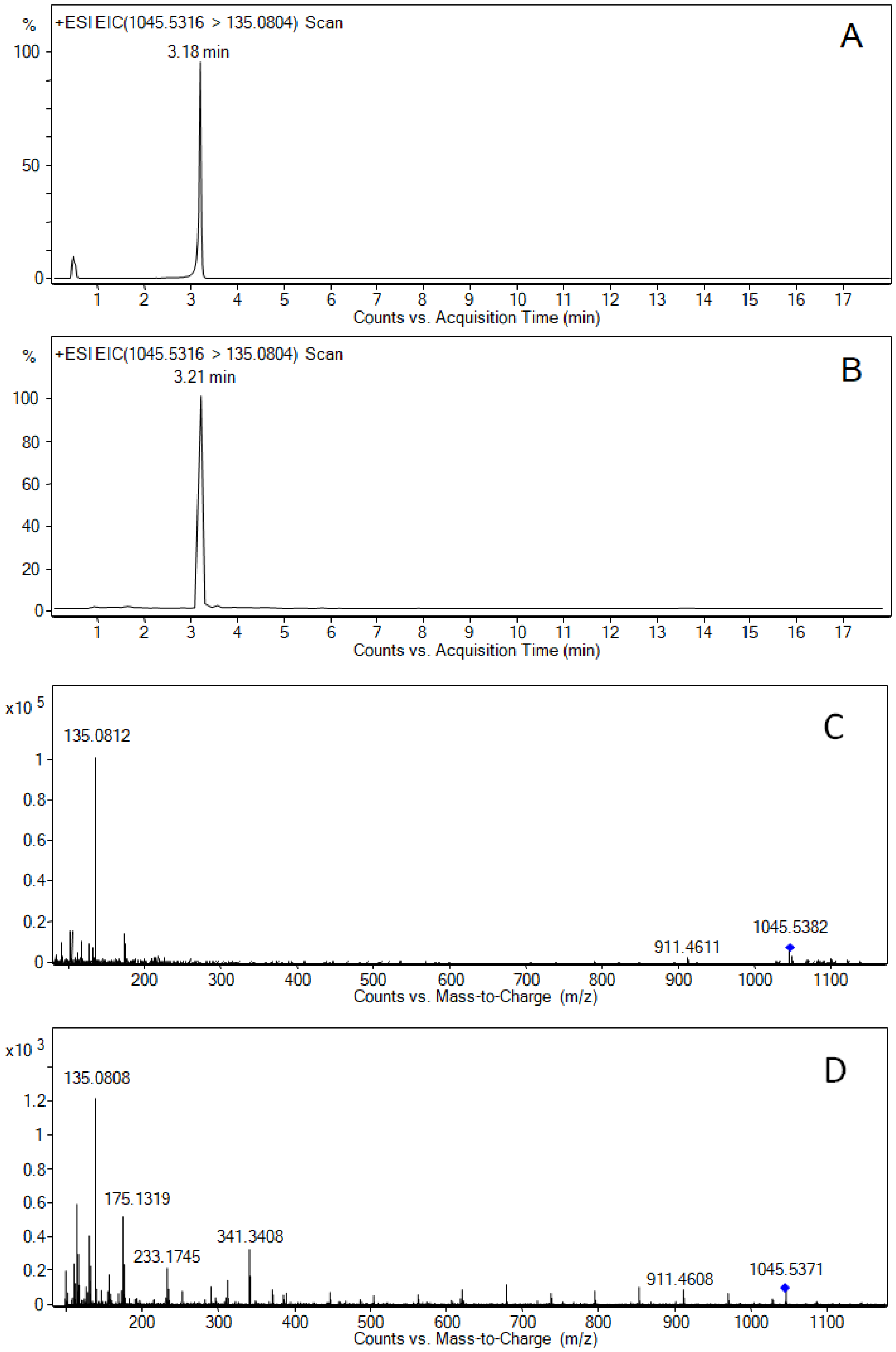

Figure S1: Extracted ion chromatograms of MC-YR calibration standard (0.1 ug/ml) (A) and *Pseudanabaena* 21.22 sample (B). Mass spectra of the transition (1045.5316 > 135.0804) corresponding to the MC-YR in the calibration standard (C) and *Pseudanabaena* 21.22 sample (D). Collision energy of 60 eV was used for the fragment transition.
